# A threshold analysis assessed the credibility of conclusions from network meta-analysis

**DOI:** 10.1016/j.jclinepi.2016.07.003

**Published:** 2016-12

**Authors:** Deborah M. Caldwell, A.E. Ades, Sofia Dias, Sarah Watkins, Tianjing Li, Nichole Taske, Bhash Naidoo, Nicky J. Welton

**Affiliations:** a School of Social and Community Medicine, University of Bristol, Canynge Hall, 39 Whatley Road, Bristol BS8 2PS, UK; b Department of Epidemiology, Johns Hopkins Bloomberg School of Public Health, 615 N Wolfe Street, Baltimore, MD 21205, USA; c Centre for Clinical Practice, National Institute for Health and Care Excellence, 10 Spring Gardens, London SW1A 2BU, UK

**Keywords:** Mixed treatment comparison, Comparative effectiveness, Health technology assessment, GRADE, Reliability, Quality assessment, Bias

## Abstract

**Objective:**

To assess the reliability of treatment recommendations based on network meta-analysis (NMA).

**Study Design and Setting:**

We consider evidence in an NMA to be potentially biased. Taking each pairwise contrast in turn, we use a structured series of threshold analyses to ask: (1) “How large would the bias in this evidence base have to be before it changed our decision?” and (2) “If the decision changed, what is the new recommendation?” We illustrate the method via two NMAs in which a Grading of Recommendations Assessment, Development and Evaluation (GRADE) assessment for NMAs has been implemented: weight loss and osteoporosis.

**Results:**

Four of the weight-loss NMA estimates were assessed as “low” and six as “moderate” quality by GRADE; for osteoporosis, six were “low,” nine were “moderate,” and 1 was “high.” The threshold analysis suggests plausible bias in 3 of 10 estimates in the weight-loss network could have changed the treatment recommendation. For osteoporosis, plausible bias in 6 of 16 estimates could change the recommendation. There was no relation between plausible bias changing a treatment recommendation and the original GRADE assessments.

**Conclusions:**

Reliability judgments on individual NMA contrasts do not help decision makers understand whether a treatment recommendation is reliable. Threshold analysis reveals whether the final recommendation is robust against plausible degrees of bias in the data.

**What is new?**

• GRADE has previously been used to assess the reliability of evidence from a network meta-analysis Quality judgements made for individual NMA contrasts do not help decision makers determine whether a given treatment recommendation is reliable.
• Health care evaluation and technology assessment organisations need to know whether potential flaws in the evidence base would change the treatment recommendation.
• A threshold analysis is used to explore how robust treatment recommendations are to plausible degrees of bias in the data.

## 1 Introduction

Network meta-analysis (NMA) is routinely used by health reimbursement agencies to evaluate the clinical and cost-effectiveness of multiple competing interventions [1], [2]. Because the statistical principles of the method are well documented [3], [4], attention has recently focused on assessing the reliability of conclusions from an NMA. Two approaches for NMA have been proposed, both based on the Grading of Recommendations Assessment, Development and Evaluation (GRADE) method for rating the confidence in an estimate from pairwise meta-analysis [5]. Briefly, a GRADE assessment rates the quality of evidence informing a pairwise meta-analysis as high, moderate, low, or very low [6] across five domains—study limitations, imprecision, indirectness, inconsistency (in the GRADE framework this is equivalent to heterogeneity), and publication bias. Evidence from randomized controlled trials starts as high confidence and can be downgraded by a maximum of two levels per domain. A summative judgment of quality is formed across all five domains [7] and interpreted as summarized in Table 1.

The GRADE working group [8] has extended this approach to NMA (GRADE NMA). Their first step is to rate confidence in each direct, pairwise summary, as mentioned previously, and the second is to generate an assessment for each of the “indirect” estimates (based on the assessment of the direct estimates informing it). For manageability, GRADE NMA focusses on “first order” indirect loops only, that is, triangular loops of three treatments A–B–C. To evaluate confidence in the effect estimates generated by the NMA, GRADE suggests using the higher of the two judgments from the direct and indirect evidence; for example, if the direct B vs. C evidence is judged as “moderate” and the indirect B vs. C as “low” quality evidence, the NMA judgment for the B vs. C contrast would be “moderate.” The process generates a set of unrelated assessments of the quality of evidence on each of the pairwise contrasts, but, critically, not on the reliability of the treatment recommendation itself.

A second proposal [9] delivers an assessment of both the confidence in the pairwise contrasts and the confidence in the treatment rankings generated by the entire network of evidence. Underlying this approach is the fact that each treatment effect estimate from an NMA is essentially a weighted average of all available direct estimates [10], [11]. These weights are used to calculate the percentage contribution of each direct estimate to each NMA estimate and, crucially, to the network as a whole [12], [13]. Then, the confidence ascribed to a treatment contrast is formed by combining the evaluation of the available direct comparisons with their relative contribution in the estimation. For pairwise estimates generated from simple networks, we expect both methods would produce similar judgments of quality.

An assessment of the quality of evidence is important; however, health care evaluation agencies also need to know whether potential problems in the evidence are serious enough that they should reconsider a treatment recommendation made on the basis of an NMA. Assuming the decision maker is interested in selecting the treatment with the highest expected efficacy, the key question regarding the quality of the NMA evidence takes the form: “given potential imperfections in the evidence, how reliable is the treatment recommendation based on the NMA?” In this article, we contrast the GRADE NMA approach with a structured series of threshold analyses that explore the robustness of a treatment recommendation to potential bias in the evidence base. We use GRADE NMA because, to date, it is the more widely implemented of the two proposals. Our starting point is the same as GRADE, namely a set of summary estimates of each of the pairwise comparisons on which there are data, and we introduce two illustrative networks to which the GRADE NMA approach has been applied. Next, we set out the principles of a threshold analysis for assessing the reliability of NMA results and apply it to the example data sets. In Section 4, we suggest alternative starting points and other extensions of the approach; we also discuss general properties of the GRADE NMA approach which may cause it to give misleading results for the decision maker.

## 2 Method

### 2.1 Illustrative examples

The illustrative examples used here are two published NMAs which have applied the assessment method proposed by the GRADE working group (GRADE NMA). Data are reported as pairwise summary estimates of treatment effect (Table 2, Table 3), and so here we assume effect estimates have arisen from two-arm trials. We consider the impact of this assumption in a sensitivity analysis. The data are described as follows:

#### 2.1.1 Branded weight-loss programs

**Fig. 1:**
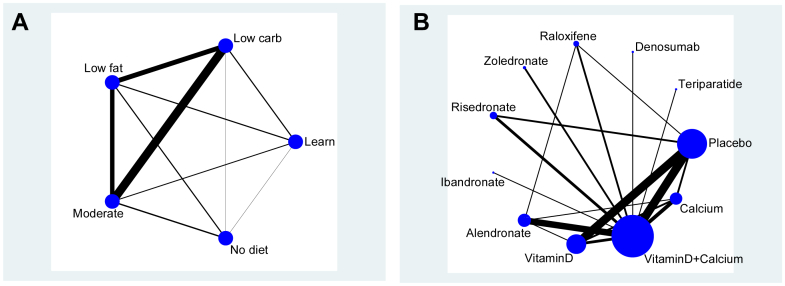
(A) Network of comparisons as described by Johnston [14]. Edge thickness is proportional to the number of trials contributing to that pairwise contrast. Treatment nodes are not weighted. (B) Osteoporosis network adapted from [8]. Edge thickness is proportional to the number of trials contributing to that pairwise contrast. Treatment nodes are proportional to number of participants.

Johnston et al. [14] analyzed branded weight-loss programs vs. another program, usual care, or no intervention. Five classes were considered: No diet, The Lifestyle, Exercise, Attitudes and Nutrition diet (LEARN), Moderate macronutrients (Moderate), Low carbohydrate (Low Carb), and Low Fat diets. Within these classes, there were total of 12 branded diets, plus LEARN and No diet. It is a fully connected network as shown in Fig. 1A, such that direct evidence was available for all 10 possible comparisons at the class level. Johnston reports mean weight loss (kg) at 12 months based on 25 trials. The data available are summarized in Table 2 in summary form for 12-month weight loss and the direct and indirect analyses and NMAs along with GRADE assessments.

#### 2.1.2 Drug treatments for osteoporosis

To illustrate the GRADE NMA approach, Puhan et al. [8] used an NMA of 10 drug treatments and placebo to prevent fractures in individuals with or at risk of osteoporosis [15]. The active treatments were alendronate, risedronate, zoledronate, ibandronate, teriparatide, raloxifene, denosumab, and calcium and/or vitamin D. Direct data, in the form of odds ratios (OR) and 95% confidence intervals (95% confidence interval), are available for 16 of the 55 possible pairwise comparisons. From Fig. 1B, we note the bulk of treatment comparisons are via placebo and vitamin D plus calcium. Four treatments are only compared to vitamin D plus calcium in single trials and are connected to the network as “spurs.” The data available are summarized in Table 3 in summary form for the outcome osteoporotic hip fracture, and the direct, indirect, and NMA analyses are shown along with the GRADE assessments.

### 2.2 Statistical analysis

#### 2.2.1 Base-case analysis

GRADE NMA assessments are performed on the summary pairwise estimates. Thus, our base-case analysis takes the form of a “two-stage” NMA [10], [16] where data are in the form of a set of summary estimates $D_{XY}$ comparing treatments X and Y with expectation $\delta_{XY}$ and variance $\sigma_{XY}^{2}$. The contribution to the likelihood is given by $D_{XY}∼N\left(\delta_{XY},\sigma_{XY}^{2}\right)$. From this “first-stage” input data set, we construct an NMA by expressing the expectations of the pairwise contrasts in terms of basic parameters $\delta_{XY}=\delta_{1Y}-\delta_{1Y},\;X,\;Y\neq 1$ which are given vague priors $\delta_{1j}∼N\left(0,100^{2}\right),\;j=2...N_{T},\;\delta_{11}=0$, where $N_{T}$ is the number of treatments. These are the effects of treatments *X* and *Y* relative to the chosen reference treatment, which is No diet in the weight-loss network and placebo in the osteoporosis network.

In both networks, the direct pairwise estimates used as inputs were based on pooled summaries from random-effects meta-analyses, as set out in the original publications [14], [15], although fixed-effect estimates were used for contrasts informed by a single trial. Global goodness of fit of the NMA can be assessed by the posterior mean of the standardized residual deviance, which will be close to the number of pairwise contrasts in a good-fitting model [17].

According to a decision-making approach, the base case recommended T∗ is the treatment with the highest expected treatment effect (or lowest depending on the context):

(1) $$T*=ArgMax_{T}{\text{E}}_{\delta }\left[\delta_{1T}\right]$$

where the vector δ represents the relative effects $\delta_{1j},\;j=2...N_{T}$. The OpenBUGS (http://www.openbugs.net/w/GNU-License) code for this base-case analysis appears in the Supplementary Materials (Section 1)/Appendix, along with the illustrative data sets.

#### 2.2.2 Threshold analysis

We compare the GRADE NMA analysis and a threshold analysis based on the two-stage NMA. The threshold analysis examines each of the summary “direct” pairwise estimates in turn and asks the following question: suppose that this summary estimate was biased, how large would the bias have to be before it led to a change in the treatment recommendation? The theory underlying this derives from the bias models familiar in both general epidemiology [18] and in bias-adjusted synthesis of RCTs [19], [20], [21], [22]. Briefly, if the available evidence $D_{XY}$ is considered to be biased, then instead of informing the target parameter $\delta_{XY}$, via $D_{XY}∼N\left(\delta_{XY},\sigma_{XY}^{2}\right)$, it informs a biased parameter $D_{XY}∼N\left(\delta_{XY}+\beta ,\sigma_{XY}^{2}\right)$. It is therefore only possible to recover an estimate of $\delta_{XY}$ from the data available to the extent that we know the distribution of the bias, $\beta ∼N\left(B,\sigma_{B}^{2}\right)$. A simple approach, then, would be to carry out a synthesis in which the original data $D_{XY}$ are replaced by a “bias-adjusted” version, approximately: $D_{XY}^{Adj}=\left(D_{XY}-\beta \right)∼N\left(\delta_{XY}-B,\sigma_{XY}^{2}+\sigma_{B}^{2}\right)$, as proposed by Turner et al. [21]. Here we assume that the bias is known, with no uncertainty ($\sigma_{B}^{2}=0$). As noted in Section 4, this represents a conservative analysis in that allowing for uncertainty would mean that larger biases were required to change the treatment recommendation.

This idea can be turned into a threshold analysis of the *XY* contrast by replacing the originally observed data $D_{XY}$ by a series of alternative values. In the analyses in the following, we explore 20 alternative values, in which $D_{XY}^{Adj}=D_{XY}\pm k\times step,\;k=1,2...10$. For example, the original mean weight change difference between Low Carb and No diet is 9.34. Accordingly, we rerun the analysis with 20 alternative values ranging from 4.34 to 14.34 in “steps” of 0.5 kg. The process is repeated for each of the pairwise contrasts on which direct evidence exists. Where the threshold is less than or equal to the step size of 0.5 kg, we specified a finer grained analysis with steps of 0.1 kg. For the osteoporosis data set where treatment effects are expressed as log odds ratios (LORs), steps of 0.5 are used initially, followed by steps of 0.1 for detailed analysis.

In each analysis, we record the goodness of fit and the new treatment T∗ with the highest posterior mean treatment effect. In a well-fitting model, the standardized posterior mean residual deviance, $\bar{D}$, should approximate the number of data points. This allows us to see whether the potential bias we are considering in each contrast is compatible with the rest of the evidence in the network. We assume that the bias is “known” with no uncertainty $\sigma_{B}^{2}=0$ (see Section 4).

OpenBUGS program code for the threshold analysis is available in the Supplementary Materials (Section 2)/Appendix.

## 3 Results

In this section, we first present the results from the GRADE NMA analyses, then the recommended treatment from the base-case two-stage NMA. This is followed by the threshold analysis and finally a relation between the GRADE NMA and threshold results.

The GRADE NMA summaries are reported in Table 2, Table 3. For the weight-loss network, overall confidence in the NMA summary effect estimates was rated as low for four comparisons and as moderate confidence for the remaining six comparisons. For the osteoporosis network, overall confidence in NMA summary effect estimates was rated as low for six, moderate for nine, and high for one comparison.

The results of the base-case two-stage NMA are summarized in Table 4. For the weight-loss network (Table 4), results suggest that a low fat weight-loss program would be preferred with the largest mean weight loss (7.88 kg) compared to No diet at 12-month follow-up. The fit of the baseline two-stage model was 11.3 compared to 10 data points, suggesting a reasonable fit of the model to the data.

The two-stage base-case analysis of the osteoporosis data (Table 4) suggests that risedronate results in the largest effect (Ln OR −1.12; standard error 0.35). Teriparatide is the second best (Ln OR − 0.87). We note that the other bisphosphonates (zoledronate, ibandronate, and alendronate), as well as denosumab, are all approximately equally effective (compared to placebo), and all have effects that are very similar to teriparatide. The fit of the baseline two-stage model was 15.3 compared to 16 data points, suggesting a good fit of the model to the data.

The results of the threshold analysis for the weight-loss network (Table 5) indicate that in 6 of the 10 contrasts, biases as large as 5 kg in either direction would make no difference to the treatment decision. In the remaining four cases, the conclusions are sensitive to potential bias. In one case, Low Carb vs. LEARN, it would be necessary to subtract 4.5 kg from the observed treatment effect to change the decision. This amount would probably be regarded as representing an implausibly large bias (+4.5 kg) in the available evidence, and even if this was not the case, the model fit statistic, 20.4 compared to 10 data points, indicates that such a bias adjustment would be incompatible with the remaining evidence. The GRADE judgment of confidence in this estimate was rated as “low”—meaning that “further research is very likely to have an important impact on our confidence in the estimate of effect and is likely to change the estimate” (see Table 1).

The base-case Low Fat decision could be changed to a Low Carb decision if the estimate of the Low Carb vs. Moderate difference (1.07) was raised by an additional kilo (downward bias, −1 kg). Positive biases (+0.9 kg) in the Low Fat vs. Moderate, or Low Far vs. Low Carb, would also lead to a change from the baseline Low Fat treatment decision to a bias-adjusted Low Carb decision. Assuming either of these two biases is present generates worse fitting models but not to an extent that rules them out on statistical grounds.

The results of the threshold analysis for osteoporosis (Table 6) indicate that in 6 of the 16 contrasts, biases as large as or larger than 2.5 in either direction on the log odds scale would make no difference to the treatment decision (risedronate). In further four cases, the conclusions are sensitive to a potential bias between 0.9 and 2.5, but these would still be regarded as an implausibly high level of bias on this scale.

For further six contrasts, the baseline treatment decision would be changed in the presence of a smaller positive bias of ±0.5 (on log odds scale). It is interesting to note that all are relative to VitD + calcium, and five of the six contrasts form spurs to the main network. The residual deviance statistics suggest that a potential bias of +0.5 for each of these contrasts is compatible with the rest of the data.

Finally, the relationship between the GRADE NMA assessments and the threshold values at which recommendations would change is set out in Table 7. For this purpose, we have considered 1 kg the largest absolute bias that would be plausible in the weight-loss data, and 0.5 on the LOR scale as the largest plausible bias in the osteoporosis data set. Based on these benchmark values, which are discussed further in the following, we find no apparent relationship between the GRADE quality assessments of NMA estimates of each contrast, and the degree to which the treatment recommendation is sensitive to bias. The results in Table 5, Table 6 can be used to construct other benchmarks, but this does not change our conclusions.

## 4 Discussion

In this article, we have set out the principles of using a threshold analysis and illustrate its potential for examining the reliability of conclusions from an NMA. In effect, the threshold analysis illustrates a form of bias correction [18], [19], [21], in which the decision maker asks, “how big must the bias be in this estimate, and in which direction, before it would lead me to change my decision? And what would the new recommendation be?” In the weight-loss example, the base-case analysis identified Low Fat as the treatment with the highest estimated effect, but it also showed that Low Carb ran a close second. The threshold analysis revealed that assumptions regarding potential bias in the most influential evidence items for the Low Fat vs. Low Carb contrast (i.e., its direct comparison and the indirect comparison via Moderate diet) were the ones where bias could impact on conclusions. Although this may not be surprising, what a threshold analysis adds is a quantification of how large a bias would need to be before a decision was changed.

The follow-up question “are biases of such a size and in such a direction plausible?” should then be answered by those with knowledge of the clinical area, and with reference to the meta-epidemiological literature on bias in trials [23], [24]. For example, in the osteoporosis network, LOR biases of 0.5 (OR 1.65) in 6 of the 16 direct estimates would change recommendations. However, application of such a high cutoff might be considered quite conservative. Hip fracture is an objectively assessable outcome and the extent of bias attributable to markers such as allocation concealment and lack of blinding has been reported to be well below that level [25]. We expect investigators would be more interested in the three direct estimates where a slightly lower level of bias (0.4, OR = 1.49), or even the single estimate where a bias of 0.3 (OR = 1.35), would be enough to change the recommendation. In two of these three, teriparatide would be the new choice, ibandronate in the other case. However, such biases are larger than those previously observed in meta-epidemiological studies for objective outcomes.

Judgments regarding bias are, of course, also subject to uncertainty regarding the size of the expected bias [26]. A comprehensive analysis of bias would, therefore, involve not only a shift in the expected effect, but a concurrent “down-weighting” of evidence and consequent increase in variance. The threshold values in Table 5, Table 6 should therefore be considered conservative; in that if we had incorporated uncertainty, it would have had the effect of increasing the degree of bias required to change the decision.

The implications of threshold analysis for decision makers are different from those generated by a GRADE assessment. The interpretation of a “low” confidence rating in GRADE is that “Further research is very likely to have an important impact on our confidence in the estimate of effect and is likely to change the estimate,” whereas a “moderate” rating suggests that “Further research is likely to have an important impact on our confidence in the estimate of effect and may change the estimate” [6]. If changing an estimate can be considered to translate into changing a decision, then one might say that threshold analysis delivers a conclusion that is diametrically opposite to GRADE because bias in the “low” confidence evidence appears to have had no consistent impact on the decision in our examples. This may not be surprising because the evaluation of “quality” in GRADE NMA is, in essence, a qualitative assessment of individual pairwise contrasts and does not take account of the overall, quantitative information flow across networks of evidence.

A limitation of our analysis is our reliance on summary data on each contrast, rather than the results of individual trials. However, by starting from exactly the same data set as the GRADE analysis, the 10 pairwise summaries for the weight-loss data and the 16 published pairwise summaries for the osteoporosis data, we have ensured a fair comparison between the threshold-based approach and GRADE for NMA. A better solution would be to run a threshold analysis within a “one-stage” framework, preferably starting from the Bayesian posterior distributions. This is because the two-stage analyses we have provided do not generate quite the same results as a one-stage NMA model. There are a number of reasons for this: some of the pairwise summaries are from fixed-effect and others from random-effects meta-analyses, whereas a one-stage NMA starting from the individual trials represents a single coherent analysis with a single random-effects variance term. Second, a correctly performed one-stage NMA automatically takes account of multiarm trials, whereas this information is lost in the pairwise contrast summaries and is not recovered in our base-case analysis. Finally, where there are zero cells in the trials, the standard pooled LOR may be quite seriously biased toward the null effect [27] and be given inaccurate precision. In the Supplementary Materials (Section 3/Appendix), we set out the differences between one- and two-stage analyses and also provide an alternative two-stage analysis of the osteoporosis data in which we use an inconsistency model [26] to stabilize between-trials variances and to maintain the correct covariance relationships for multiarm trials. The results (Table S2/Appendix at www.jclinepi.com) suggest that, with these improvements, a two-stage analysis delivers base-case results that are almost identical to a one-stage NMA.

A further weakness of our analysis, but one which equally applies to the GRADE approach, is that each pairwise comparison and the potential biases attached to it are considered independent of all the others. For example, if we consider the GRADE domains “study limitations” and “publication bias,” it is likely that within the entire evidence ensemble, several studies will be vulnerable to precisely the same kinds of bias, operating to the same or similar extent, and in the same direction [28], [29], [30], [31], [32], [33]. We have also only considered that a single contrast can be biased. In practice, it is more likely that several treatment contrasts in a network would be vulnerable to the same potential biases, for example, if participants are unable to be blinded to a treatment to which several others are compared. Joint modeling of such biases would be preferable. In many cases, perhaps especially with subjective outcomes, we suggest that the base-case analysis should not be a standard NMA, but a bias-adjusted NMA. The threshold analyses we are proposing here could be extended further to apply to such bias-adjustment models, although we note that this goes beyond the intentions of the GRADE framework which is to assess confidence in the results of meta-analysis, rather than make adjustments to a meta-analysis.

A further possibility would be to combine a threshold analysis and risk of bias assessment to give a reliability rating similar in spirit to GRADE but driven by sensitivity to bias as well as the likelihood of bias. We would, however, caution against reliability ratings as they fail to provide nuanced recommendations for clinicians. For example, rather than assign a rating of moderate quality evidence to a Low Fat diet, it is more useful to recommend Low Fat but to add that a Low Carb diet could also be confidently recommended to patients who do not want to try Low Fat. This is based on both the threshold analysis and the fact that Low Carb was a close second in the NMA.

Both GRADE NMA and the threshold analysis allow for an explicit and systematic approach to facilitate informed decision making. The essential difference is that the former provides a set of unrelated quality assessments of the estimate for each contrast, which, in the two examples we have examined, show no relationship to the reliability of the treatment recommendation, when interpreted as its sensitivity to bias. The threshold analysis provides the decision maker with clear information on the extent to which a recommendation might be vulnerable to potential biases in the evidence. Identification of items of data to which the decision is sensitive could also be used to inform future research needs, particularly where the item is also at high risk of bias. Further statistical work is in progress to develop the threshold analysis so the starting point is the posterior distribution delivered by a Bayesian one-stage NMA, as this will allow complete flexibility in the face of the complex and irregular forms of evidence usually encountered in practice. It is also important to ensure that the threshold analysis can be applied to different objective functions that decision makers might use, such as multicriterion decision analysis [34], [35] and Net Benefit analysis [36].

## Figures and Tables

**Table 1:** Levels of quality assigned by the GRADE approach to assessing the confidence that can be assigned to the pooled effect estimate from a pairwise meta-analysis

Quality level	Current definition	Previous definition
High	We are very confident that the true effect lies close to that of the estimate of the effect	Further research is very unlikely to change our confidence in the estimate of effect
Moderate	We are moderately confident in the effect estimate: The true effect is likely to be close to the estimate of the effect, but there is a possibility that it is substantially different	Further research is likely to have an important impact on our confidence in the estimate of effect and may change the estimate
Low	Our confidence in the effect estimate is limited: The true effect may be substantially different from the estimate of the effect	Further research is very likely to have an important impact on our confidence in the estimate of effect and is likely to change the estimate
Very low	We have very little confidence in the effect estimate: The true effect is likely to be substantially different from the estimate of effect	Any estimate of effect is very uncertain

*Abbreviation:* GRADE, Grading of Recommendations Assessment, Development and Evaluation.

**Table 2:** Weight-loss programs: summary results (difference in mean weight loss [kg]) and GRADE assessment of the direct and indirect analyses and NMAs

Comparison (active vs. control)	Mean difference (95% CI)			Quality of evidence (GRADE)		
	Direct	Indirect	Network	Direct	Indirect	Network
LEARN vs. No diet	3.67 (−3.88, 11.21)	3.63 (0.36, 6.91)	5.16 (2.68, 7.63)	Low	Low	Low
Moderate vs. No diet	4.84 (2.82, 6.86)	4.69 (1.73, 7.75)	5.70 (4.14, 7.35)	Low	Low	Moderate
Low Carb vs. No diet	9.34 (7.31, 11.37)	5.16 (2.25, 8.18)	7.25 (5.33, 9.25)	Low	Moderate	Moderate
Low fat vs. No diet	5.97 (2.01, 9.92)	6.15 (2.96, 9.40)	7.27 (5.26, 9.34)	Moderate	Moderate	Moderate
Moderate vs. LEARN	0.21 (−4.64, 5.05)	0.94 (−1.74, 3.66)	0.55 (−1.71, 2.87)	Low	Low	Low
Low Carb vs. LEARN	1.23 (−1.22, 3.67)	2.48 (−0.19, 5.19)	2.10 (−0.20, 4.47)	Low	Low	Low
Low fat vs. LEARN	4.00 (−0.21, 8.21)	2.64 (−0.02, 5.33)	2.12 (−0.33, 4.59)	Low	Low	Low
Low Carb vs. Moderate	1.07 (0.16, 1.97)	2.05 (−0.92, 4.96)	1.55 (0.13, 2.95)	Moderate	Low	Moderate
Low fat vs. Moderate	1.84 (0.96, 2.72)	1.38 (−0.75, 3.51)	1.56 (−0.17, 3.30)	Moderate	Low	Moderate
Low fat vs. Low Carb	0.33 (−0.86, 1.52)	0.39 (−1.92, 2.70)	0.02 (−1.78, 1.79)	Low	Moderate	Moderate

*Abbreviations:* GRADE, Grading of Recommendations Assessment, Development and Evaluation; NMAs, network meta-analyses; CI, confidence interval.

**Table 3:** Osteoporotic hip fractures GRADE NMA assessment of the direct and indirect analyses and NMAs

Comparison (active vs. control)	Odds ratio (95% CI)			Quality of evidence (GRADE)		
	Direct	Indirect	Network	Direct	Indirect	Network
Raloxifene vs. placebo	0.84 (0.63 to 1.13)	0.96 (0.53 to 1.78)	0.87 (0.63 to 1.22)	Moderate	Low	Moderate
Risedronate vs. placebo	0.17 (0.05 to 0.59)	0.54 (0.36 to 0.75)	0.48 (0.31 to 0.66)	Low	Low	Low
Vitamin D vs. placebo	1.25 (0.82 to 1.89)	1.08 (0.61 to 1.91)	1.13 (0.94 to 1.34)	Low	Low	Low
Vitamin D + calcium vs. placebo	0.83 (0.73 to 0.96)	0.54 (0.29 to 0.94)	0.81 (0.68 to 0.96)	Moderate	Low	Moderate
Vitamin D + calcium vs. teriparatide	2.00 (0.50 to 8.33)	—	1.92 (0.45 to 8.42)	Low	—	Low
Vitamin D + calcium vs. denosumab	1.67 (1.02 to 2.70)	—	1.64 (0.97 to 2.87)	Moderate	—	Moderate
Alendronate vs. raloxifene	0.49 (0.04 to 5.45)	0.53 (0.30 to 0.90)	0.51 (0.29 to 0.87)	Low	Moderate	Moderate
Vitamin D + calcium vs. raloxifene	0.88 (0.51 to 1.54)	0.96 (0.63 to 1.49)	0.94 (0.66 to 1.31)	Moderate	Low	Moderate
Vitamin D + calcium vs. zoledronate	1.64 (1.16 to 2.17)	—	1.63 (1.16 to 2.30)	High	—	High
Vitamin D + calcium vs. risedronate	1.92 (0.84 to 4.35)	5.88 (1.79 to 25.00)	1.69 (1.27 to 2.54)	Very low	Low	Low
Vitamin D + calcium vs. ibandronate	1.72 (0.76 to 3.85)	—	1.69 (0.69 to 3.84)	Low	—	Low
Vitamin D vs. alendronate	3.70 (1.20 to 11.11)	2.38 (1.49 to 3.85)	2.54 (1.63 to 4.16)	Moderate	Moderate	Moderate
Vitamin D + calcium vs. alendronate	1.59 (1.03 to 2.44)	2.78 (1.14 to 8.33)	1.82 (1.24 to 2.90)	Moderate	Moderate	Moderate
Calcium vs. alendronate	4.55 (0.47 to 50.00)	2.56 (1.54 to 4.35)	2.56 (1.57 to 4.34)	Very low	Moderate	Moderate
Vitamin D + calcium vs. vitamin D	1.03 (0.68 to 1.54)	0.65 (0.48 to 0.85)	0.72 (0.57 to 0.91)	Low	Low	Low
Calcium vs. calcium + vitamin D	1.21 (0.89 to 1.66)	3.43 (0.26 to 160.4)	1.40 (1.03 to 1.95)	Low	Very low	Moderate

*Abbreviations:* GRADE, Grading of Recommendations Assessment, Development and Evaluation; NMAs, network meta-analyses; CI, confidence interval.

Estimates are odds ratios (ORs), where OR <1 favors active treatment.

**Table 4:** Base-case NMA based on the two-stage method, posterior summaries

Treatment	Pr(Best)	Treatment effect estimate	SD
Branded weight-loss programs			
No diet	0	Reference	—
LEARN	0.01	5.56	1.16
Moderate	0	6.09	0.72
Low Carb	0.17	7.49	0.72
**Low Fat**	**0.82**	**7.88**	**0.76**
Hip fracture treatments in osteoporosis			
Placebo	0.00	Reference	—
Teriparatide	0.32	−0.87	0.72
Denosumab	0.04	−0.69	0.26
Raloxifene	0.00	−0.15	0.13
Zoledronate	0.02	−0.68	0.17
**Risedronate**	**0.45**	**−1.12**	**0.35**
Ibandronate	0.12	−0.72	0.42
Alendronate	0.05	−0.75	0.21
Vitamin D	0.00	0.04	0.15
Vitamin D + calcium	0.00	−0.18	0.07
Calcium	0.00	0.02	0.17

*Abbreviations:* NMA, network meta-analysis; SD, standard deviation.

The entries in bold indicate the treatment, which would be recommended on the base-case analysis, and is the one with the highest ranked mean treatment effect.

The treatment effect estimate for the branded weight loss programs is mean kg difference. For Hip fracture it is the odds ratio.

**Table 5:** Threshold analysis for branded weight-loss programs [14] new recommended treatment, threshold at which new recommendation is made, and posterior residual mean deviance of the adjusted data NMA model at the threshold adjustment

Treatment B (active)	Treatment A (control)	Estimate (B relative to A)	SE	Trials	Recommendation	Bias threshold, kg	Deviance	GRADE NMA
LEARN	No diet	3.67	3.85	2	n.c	n.f	—	Low
Moderate	No diet	4.84	1.03	7	n.c	n.f	—	Moderate
Low Carb	No diet	9.34	1.04	1	n.c	n.f	—	Moderate
Low fat	No diet	5.97	2.02	3	n.c	n.f	—	Moderate
Moderate	LEARN	0.21	2.47	2	n.c	n.f	—	Low
Low Carb	LEARN	1.23	1.25	2	LEARN	4.5	20.4	Low
Low fat	LEARN	4.00	2.15	2	n.c	n.f	—	Low
Low Carb	Moderate	1.07	0.46	10	**Low Carb**	**−1.0**	9.9	Moderate
Low fat	Moderate	1.84	0.45	4	**Low Carb**	**+0.9**	12.1	Moderate
Low fat	Low Carb	0.33	0.61	4	**Low Carb**	**+0.9**	13.0	Moderate

*Abbreviations:* NMA, network meta-analysis; SE, standard error; GRADE, Grading of Recommendations Assessment, Development and Evaluation; n.c., no change; n.f., not found.

Original treatment decision based on two-stage NMA was Low Fat. Entries in bold indicate evidence sources in which a plausible bias could change the treatment decision from Low Fat. For example, if the pairwise evidence Low Carb vs. Moderate was subject to a bias of −1 kg or more (i.e., the unbiased estimate was not the observed 1.07 kg, but 2.07 kg or higher), the treatment recommendation would change to Low Carb. n.c. indicates no change in recommended treatment. n.f. indicates that no threshold was found within ±5 kg. The GRADE assessment is from the last column in Table 2.

**Table 6:** Threshold analysis for drug treatments to prevent osteoporotic hip fractures [8], [15]: new recommended treatment, threshold at which new recommendation is made and posterior residual mean deviance of the adjusted data NMA model at the threshold adjustment

Treatment B (active)	Treatment A (control)	LOR	SE	Trials	Recommendation	Bias threshold (LOR)	Deviance	GRADE NMA
		B relative to A						
Raloxifene	Placebo	−0.17	0.24	1	Raloxifene	−1.5	41.0	Moderate
Risedronate	Placebo	−1.77	0.24	2	**Teriparatide**	**+0.9**	13.8	Low
Vitamin D	Placebo	0.22	0.69	9	Vitamin D	−3	92.1	Low
Vitamin D + calcium	Placebo	−0.19	0.04	8	Teriparatide	−1	39.0	Moderate
Vitamin D + calcium	Teriparatide	0.69	2.30	1	**Teriparatide**	**+0.3**	15.3	Low
Vitamin D + calcium	Denosumab	0.51	0.99	1	**Denosumab**	**+0.5**	15.3	Moderate
Alendronate	Raloxifene	−0.71	2.52	1	n.c	n.f	—	Moderate
Vitamin D + calcium	Raloxifene	−0.13	0.60	2	Raloxifene	+4.5	196.0	Moderate
Vitamin D + calcium	Zoledronate	0.49	0.74	2	**Zoledronate**	**+0.5**	15.3	High
Vitamin D + calcium	Risedronate	0.65	1.51	3	**Teriperatide**	**−0.4**	16.9	Low
Vitamin D + calcium	Ibandronate	0.54	1.42	1	**Ibandronate**	**+0.4**	15.3	Low
Vitamin D	Alendronate	1.31	2.36	1	Alendronate	+3.5	58.0	Moderate
Vitamin D + calcium	Alendronate	0.46	0.88	7	**Alendronate**	**+0.5**	14.0	Moderate
Calcium	Alendronate	1.52	4.10	1	n.c	n.f	—	Moderate
Vitamin D + calcium	Vitamin D	0.03	0.53	2	Vitamin D	+2.5	115.2	Low
Calcium	Vitamin D + calcium	0.19	0.54	4	Calcium	−1.5	18.5	Moderate

*Abbreviations:* LOR, log odds ratio; SE, standard error; GRADE, Grading of Recommendations Assessment, Development and Evaluation; NMA, network meta-analysis; n.c., no change; n.f., not found.

Original treatment decision based on two-stage analysis was risedronate. Entries in bold indicate evidence sources in which a plausible bias could change the treatment decision from risedronate. For example, consider the pairwise (direct) evidence on placebo vs. risedronate, if the pairwise evidence was subject to a bias of +0.9 on the log odds ratio scale or more (i.e., the unbiased estimate was not the observed −1.77, but was −0.87 or higher), the treatment recommendation would change to teriparatide. The GRADE NMA assessment is from the last column in Table 3. n.c. indicates no change in recommended treatment. n.f. indicates no threshold was found within ±5 on a log odds ratio scale.

**Table 7:** Relationship between distribution of GRADE NMA quality assessments and the contrasts to which recommendations are sensitive

Example dataset	High	Moderate	Low	Very low	Total
Weight loss					
All contrasts	0	6	4	0	10
Sensitive contrasts	0	3	0	0	3
Osteoporosis					
All contrasts	1	9	6	0	16
Sensitive contrasts	1	2	4	0	7

*Abbreviations:* GRADE, Grading of Recommendations Assessment, Development and Evaluation; NMA, network meta-analysis.

## Supplementary data

Supplementary Materials (Sections 1–3) and Tables S1 and S2
