# Supplementary material for: A threshold analysis assessed the credibility of conclusions from network meta-analysis
Source: J Clin Epidemiol. 2016 Dec;80:68–76. doi: 10.1016/j.jclinepi.2016.07.003 (PMC5176010; doi:10.1016/j.jclinepi.2016.07.003)
Supplement: Supplementary Materials (Sections 1–3) and Tables S1 and S2 [file mmc1.pdf]

SUPPLEMENTARY MATERIALS:

Threshold analysis to assess the credibility of conclusions from network meta-analysis

1. WinBUGS code for the base-case 2-stage NMA

The base-case two-stage analysis is as follows. The input data are the summary relative effects based on separate meta-analyses of each of the pair-wise contrasts. The weight-loss and osteoporosis datasets appear below

```
model {                              # base case analysis
d[1]  <- 0
for (k in 2:NT) { d[k] ~ dnorm(0,0.0001)  }                        # priors for NT-1 basic parameters
for (c in 1:(NT-1))  { for (k in (c+1): NT)  {  delta[c,k] <- d[k] - d[c]          # model the NT*(NT-1)/2 contrasts
for (i in 1:N) { D[i] ~ dnorm(delta[b[i],t[i]],P[i])                # likelihood
                 dev[i] <- P[i] * pow((D[i]-delta[b[i],t[i]]),2) }  # deviance
sumdev <- sum(dev[])                                                 # summed deviance
}
```

#Weight-loss pair-wise inputs

list(NT=5,N=10)

t[]	b[]	D[]	P[]
2	1	3.67	0.067482887
3	1	4.84	0.941476326
4	1	9.34	0.932223543
5	1	5.97	0.2455948
3	2	0.21	0.163653229
4	2	1.23	0.642620263
5	2	4.00	0.216744433
4	3	1.07	4.690455114
5	3	1.84	4.960743802
5	4	0.33	2.712802768

# Osteoporosis pair-wise inputs

list(NT=11,N=16)

t[]	b[]	D[]	P[]
4	1	-0.174353387	45.01633091
6	1	-1.771956842	2.522590727
9	1	0.223143551	22.0379046
10	1	-0.186329578	204.8442346
10	2	0.693147181	1.941911284
10	3	0.512823626	16.21606951
8	4	-0.713349888	0.63623121
10	4	-0.127833372	12.5819449
10	5	0.494696242	39.17395115
10	6	0.652325186	5.6818392
10	7	0.542324291	5.837103216
9	8	1.30833282	3.102469721
10	8	0.463734016	20.65927709
11	8	1.515127233	0.705485428
10	9	0.029558802	22.99615142
11	10	0.19062036	39.54633397

2. Threshold analysis

The threshold analysis is achieved by the following code, using the same datasets as above, but replacing the "list" input by, for example, list(NT=5,N=10, Nstep=10, K=9, inc=0.5) for the weight loss data. This will have the effect of carrying out a threshold analysis on data element 9, in 10 steps of 0.5 kg each above, and 10 steps below, the original data for the weight loss data.

```
model {                                    #    threshold analysis
for (h in 1: (2*Nstep +1) )  {                                  #  loop over h = 2*Nstep+1 settings of bias in pooled
summary K
d[h,1]  <- 0
for (k in 2:NT) { d[h,k] ~ dnorm(0,0.0001)  }                   # priors for NT-1 basic parameters
for (c in 1:(NT-1))  { for (k in (c+1): NT)  {  dd[h,c,k] <- d[h,k] - d[h,c] }}        # model the NT*(NT-1)/2

DH[h,K] <- D[K] + (h-Nstep-1) *inc                              # construct alternative datasets DH[h,] from data D[]
for (i in 1:(K-1)) { DH[h,i] <- D[i] }                          # except for data element D[K] set the rest of DH[h,] to D[]
for (i in (K+1):N) { DH[h,i] <- D[i] }                          #                              ditto

for (i in 1:N) { DH[h,i] ~ dnorm(dd[h,b[i],t[i]],P[i])          # likelihood
       dev[h,i] <- P[i] * pow((DH[h,i]-dd[h,b[i],t[i]]),2) }    # deviance
sumdev[h] <- sum(dev[h,])                                       # summed deviance
}                                                               # end loop h
}
```

3: Comparisons of original one-stage NMA and two-stage NMA

For the weight loss network we compare the two-stage analysis used as our base case with the one-stage Bayesian NMA as reported (Table S1). Both analyses would lead to the same treatment recommendation (Low Fat) although there are noticeable differences between the two NMAs

For the osteoporosis data we compare the results as reported for the original one-stage Bayesian random effects NMA (Table S2 columns 7,8,9) with two forms of two-stage NMA . The first, used in the paper, is based on the separate pair-wise summaries reported in the original publication (Table 2 columns 1,2,3). The second (Table 2 columns 4,5,6) is based on the output from Bayesian random effects meta-analysis of each of the separate pair-wise contrasts subject to the restriction that they all share the same between-trials variance [1]. This analysis requires the same original trial-level data that is used as input to a one-stage NMA. The effect is to stabilise the variances of each contrast. In addition the posterior correlations between-treatment effects in multi-arm trials, is carried through to the second stage. The 2nd-stage NMA (not shown here) is modified to take account of the correlation structure

The results (Table S2 columns 4,5,6) show that the alternative two-stage analysis with stabilised variances and correlations produces results that are virtually indistinguishable from a standard one-stage Bayesian NMA.

Our interpretation of this is that, if a two stage approach is to be used for threshold analysis, the stabilised variance and correlations approach is preferable, as the base-case results will be very much closer to the results of the one-stage NMA on which recommendations will be based.

Table S1. Comparison of two-stage NMA starting from crude pair-wise summaries, and one-stage Bayesian RE NMA for the branded weight loss programs [2]

	Two-Stage analysis			Original one-stage analysis	
**Treatment**	**Pr(Best)**	**Mean kg difference**	**SD**	**Mean kg difference**	**SD**
No diet	0	(reference)	-	(reference)	
LEARN	0.01	5.56	1.16	5.16	1.26
Moderate	0	6.09	0.72	5.70	0.81
Low CHO	0.17	7.49	0.72	7.25	0.99
Low Fat	0.82	7.88	0.76	7.27	1.04

Table S2. Comparing the two stage base case analysis for osteoporosis with a modified two stage NMA with stabilised variances and incorporating correlations, and the original one stage analysis as published by Murad *et al*. [3] [4]

**Treatment**	**Two-Stage analysis based on pair-wise summaries**			**Two stage analysis with stabilised variance and correlations**			**Original one-stage analysis (as published)**		
	Pr(Best)	**LOR**	**SD**	***Pr(Best)***	**LOR**	**SD**	***Pr(Best)***	**LOR**	**SD**
**Placebo**	*0*	-	-	*0.00*	-	-	*0.00*	-	-
**Teriparatide**	*0.32*	-0.87	0.72	*0.41*	-0.89	0.76	*0.42*	-0.87	0.74
**Denosumab**	*0.04*	-0.69	0.26	*0.12*	-0.74	0.30	*0.13*	-0.69	0.30
**Raloxifene**	*0.00*	-0.15	0.13	*0.00*	-0.13	0.17	*0.00*	-0.14	0.17
**Zoledronate**	*0.02*	-0.68	0.17	*0.05*	-0.70	0.20	*0.05*	-0.69	0.19
**Risedronate**	*0.45*	-1.12	0.35	*0.07*	-0.74	0.19	*0.06*	-0.73	0.19
**Ibandronate**	*0.12*	-0.72	0.42	*0.20*	-0.75	0.45	*0.21*	-0.71	0.44
**Alendronate**	*0.05*	-0.75	0.21	*0.15*	-0.83	0.23	*0.14*	-0.80	0.24
**VitD**	*0.00*	0.04	0.15	*0.00*	0.12	0.09	*0.00*	0.12	0.09
**VitD+Calcium**	*0.00*	-0.18	0.07	*0.00*	-0.22	0.09	*0.00*	-0.21	0.09
**Calcium**	*0.00*	0.02	0.17	*0.00*	0.15	0.18	*0.00*	0.13	0.17

1. Dias, S., et al., *NICE DSU Technical Support Document 4: Inconsistency in networks of evidence based on randomised controlled trials*, in *Technical Support Document*. 2011.
2. Johnston, B.C., et al., *Comparison of weight loss among named diet programs in overweight and obese adults: A meta-analysis.* JAMA, 2014. **312**(9): p. 923-933.
3. Murad, M.H., et al., *Comparative Effectiveness of Drug Treatments to Prevent Fragility Fractures: A Systematic Review and Network Meta-Analysis.* The Journal of Clinical Endocrinology & Metabolism, 2012. **97**(6): p. 1871-1880.
4. Puhan, M.A., et al., *A GRADE Working Group approach for rating the quality of treatment effect estimates from network meta-analysis.* BMJ, 2014. **349**: p. g5630.
